# Neuroendocrine neoplasm of the minor papilla diagnosed with endoscopic ultrasonography‐guided fine‐needle biopsy and curatively resected by endoscopic papillectomy

**DOI:** 10.1111/den.14953

**Published:** 2024-11-07

**Authors:** Kento Shionoya, Kenjiro Yamamoto, Takao Itoi

**Affiliations:** ^1^ Department Gastroenterology and Hepatology Tokyo Medical University Tokyo Japan

## Abstract

Watch a video of this article.

## BRIEF EXPLANATION

Minor papillary neoplasms are rare and surgical resection is the most reported treatment.^1^ Moreover, reports of endoscopic resection of neoplasm in the minor papilla are scarce.^2^

**Figure 1:**
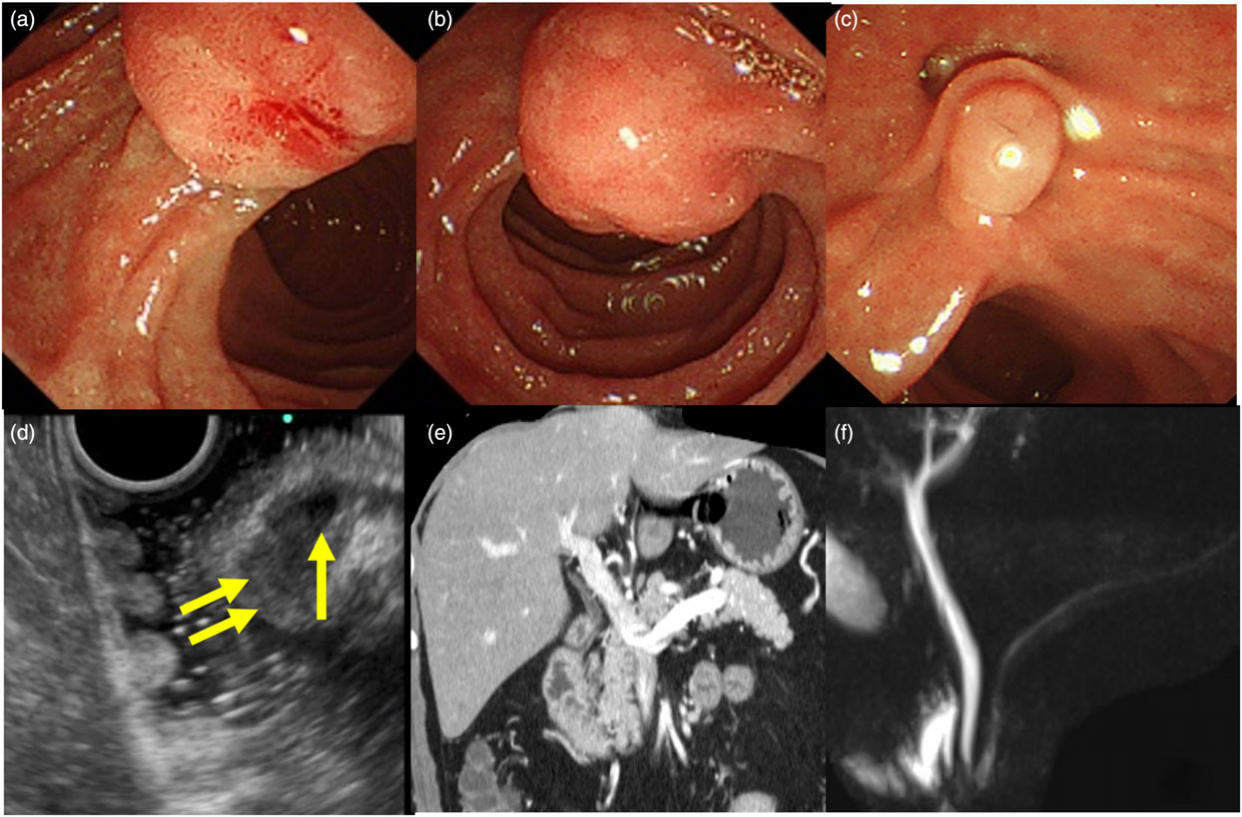
Abdominal examination images. (a) Upper gastrointestinal endoscopy showing an enlarged view to about 10 mm minor papilla. (b) Duodenoscopy showing a submucosal epithelial lesion in the minor papilla. (c) Duodenoscopy showing the major papilla, but no significant changes were noted. (d) Endoscopic ultrasonography showing an 8 mm hypoechoic neoplasm within the submucosal layer. There is dilation of the duct of Santorini, without invasion of the muscularis propria or intraductal extension to the pancreatic duct (arrow) showed dilation of the duct of Santorini and (double arrow) showed a hypoechoic neoplasm. (e) Abdominal computed tomography showing no distant metastasis. (f) Magnetic resonance cholangiopancreatography does not show pancreatic divisum.

**Figure 2:**
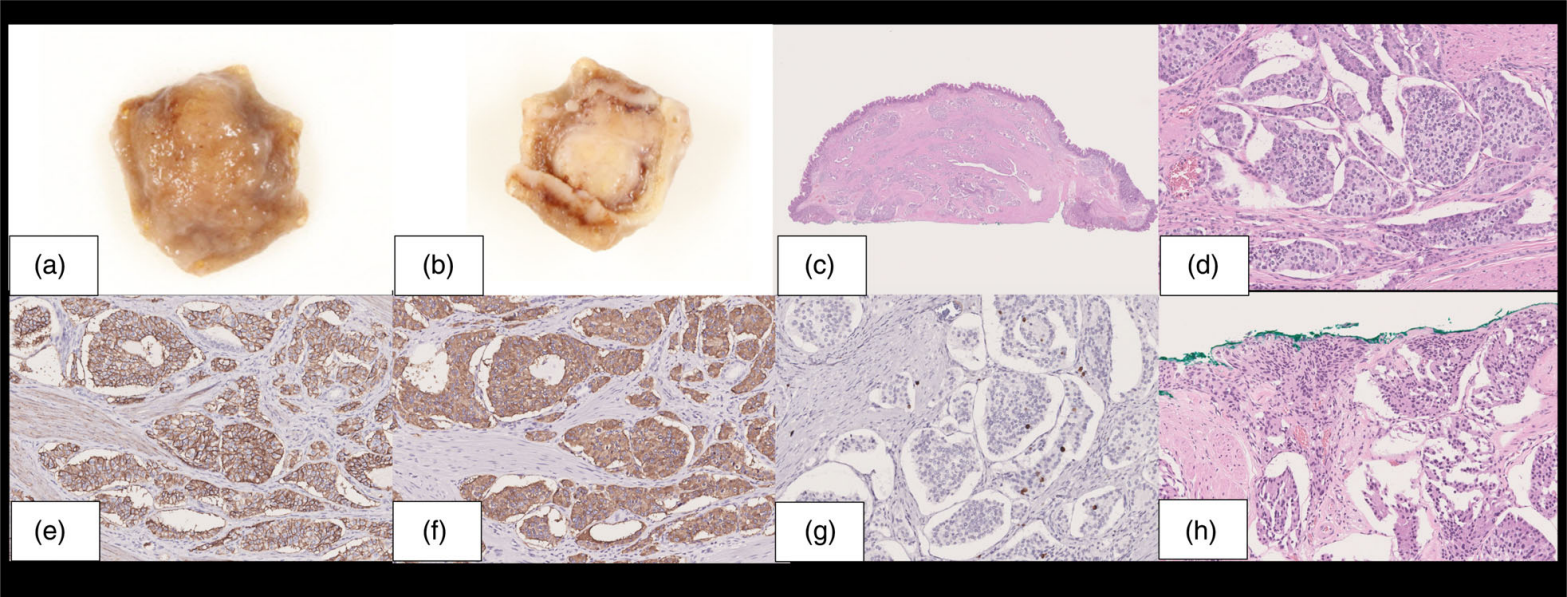
Histological findings of the endoscopic papillectomy (EP) for neuroendocrine neoplasm of the minor papilla. (a) Macroscopic image of the neoplasm (mucosal side). (b) Macroscopic image of the neoplasm (opposite side). (c) Low‐magnification image of neoplasm stained with hematoxylin and eosin. The tumor was completely resected. (d) High‐magnification image of neoplasm stained with hematoxylin and eosin. There was no invasion of the muscularis propria or lymphovascular invasion. (e) CD56 staining image. CD56 staining was positive. (f) Synaptophysin staining image. Synaptophysin staining was positive. (g) Ki‐67 index. Ki‐67 index was 1% positive. (h) Moderate magnification of hematoxylin and eosin‐stained neoplasm slides. There was no invasion of the muscularis propria or lymphovascular invasion.

A 47‐year‐old man with an enlarged minor papilla detected on upper gastrointestinal endoscopy was referred to our institution (Fig. 1a). Duodenoscopy revealed a submucosal epithelial lesion in the minor papilla (Fig. 1b), and endoscopic ultrasonography (EUS) showed an 8 mm hypoechoic neoplasm within the submucosal layer without invasion of the muscularis propria or intraductal extension into the pancreatic duct (Fig. 1c,d). Contrast‐enhanced EUS showed that the neoplasm was contrast on isoechoic. Based on EUS‐guided fine‐needle biopsy (EUS‐FNB) with a 22G three‐prong asymmetry tip needle (Trident; Micro‐Tech Endoscopy, Nanjing, China) using the fanning technique, the lesion was diagnosed as a low‐grade (G1) neuroendocrine neoplasm (NEN). Computed tomography and magnetic resonance cholangiopancreatography showed no distant metastases or pancreatic divisum (Fig. 1e,f). The patient declined surgery, so endoscopic papillectomy (EP) was performed. The scope was placed in a semi‐push position to position the lesion favorably. A snare was placed on the oral side of the lesion, which was then grasped by pushing the snare inward. During grasping, the scope was placed in a pulled position by stretching. The lesion was resected en bloc in endocut mode. Subsequently, pulsatile bleeding was controlled using hemostatic clips. A pancreatic ductal stent was not placed, as the pancreatic divisum was absent (Video S1). The pathological diagnosis was NEN‐G1 without invasion of the muscularis propria or lymphovascular invasion, and the neoplasm was completely resected without any complications (Fig. 2). There was no recurrence within 1 year.

EUS‐FNB can be used to diagnose NEN of the minor papilla. EP can be effective for NEN of the minor papilla and should be considered when the neoplasm is <10 mm without intrinsic muscle layer invasion or lymph node metastasis.^3^

Authors declare no conflict of interest for this article.

## ETHICS STATEMENT

Approval of the research protocol by an Institutional Reviewer Board: N/A.

Informed Consent: Informed consent was obtained from the patient in this case report.

Registry and the Registration No. of the study/trial: N/A.

Animal Studies: N/A.

## Supporting information


**Video S1** Endoscopic papillectomy for a neuroendocrine neoplasm of the minor papilla was performed.
